# Automated assembly of species metabolomes through data submission into a public repository

**DOI:** 10.1093/gigascience/gix062

**Published:** 2017-07-19

**Authors:** Reza M. Salek, Pablo Conesa, Keeva Cochrane, Kenneth Haug, Mark Williams, Namrata Kale, Pablo Moreno, Kalai Vanii Jayaseelan, Jose Ramon Macias, Venkata chandrasekhar Nainala, Robert D. Hall, Laura K. Reed, Mark R. Viant, Claire O’Donovan, Christoph Steinbeck

**Affiliations:** 1 European Molecular Biology Laboratory, European Bioinformatics Institute (EMBL-EBI), Wellcome Trust Genome Campus, Hinxton, Cambridge CB10 1SD, UK; 2 Department of Biological Sciences, University of Alabama, P.O. Box 870344, Tuscaloosa, AL 35487, USA; 3 School of Biosciences, University of Birmingham, Birmingham B15 2TT, UK; 4 Wageningen University and Research, Wageningen Plant Research - Bioscience, P.O. Box 16, 6700AA, Wageningen, the Netherlands; 5 Institute for Inorganic and Analytical Chemistry, Friedrich-Schiller-University, Lessingstr. 8, 07743 Jena, Germany

**Keywords:** metabolomics, databases, curation, species metabolomes

## Abstract

Following similar global efforts to exchange genomic and other biomedical data, global databases in metabolomics have now been established. MetaboLights, the first general purpose, publically available, cross-species, cross-application database in metabolomics, has become the fastest growing data repository at the European Bioinformatics Institute in terms of data volume. Here we present the automated assembly of species metabolomes in MetaboLights, a crucial reference for chemical biology, which is growing through user submissions.

## Background

Following data standardization efforts in the 1990s and the success of global efforts to exchange genomic [1, 2], proteomic [3], gene expression [4], and other biomedical data, we have now witnessed the emergence of global databases in metabolomics.

In 2012, the European Bioinformatics Institute launched MetaboLights (RRID:SCR_014663) [5, 6], the first general purpose, cross-species, cross-application database in metabolomics, aiming at a similar growth in this remaining large pillar of ‘omics sciences [7]. Within the first 2 years of its inception, MetaboLights became the fastest growing data repository at the European Bioinformatics Institute (EMBL-EBI) in terms of data volume. Here we present the automated assembly and growth of species metabolomes in the MetaboLights reference layer, which is largely driven by user submissions. Journals already demand or recommend the deposition of metabolomics studies in MetaboLights. These include *Nature, EMBO, PLoS*, BioMed Central, *Frontiers, Metabolomics*, and *MDPI Metabolites*. To the best of our knowledge, MetaboLights is the only global, general purpose repository that systematically requires the submission of a metabolites assignment, a requirement fundamental for the process described here.

## Findings

**Figure 1:**
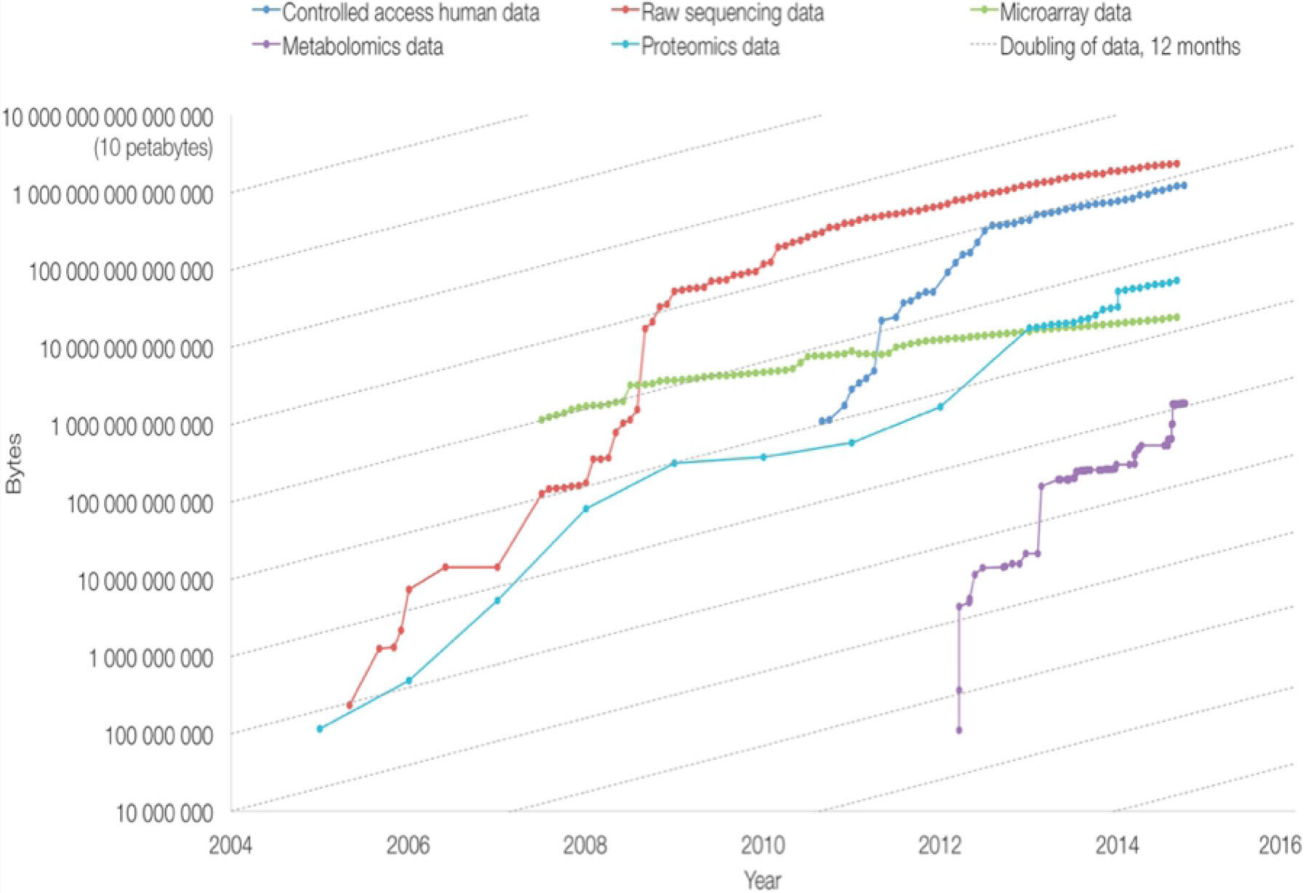
Growth in data repositories at the EMBL-EBI. The graph shows the data volume in each of the repositories over time on a logarithmic scale. Shown are repositories for controlled access human data, raw sequencing data, microarray, proteomics, and metabolomics data. Archives were started at different moments in history. Metabolomics shows the steepest growth of all repositories at the EMBL-EBI.

A fundamental, unsolved problem in Metabolomics is the availability of exhaustive model organism metabolomes. The newly formed Model Organism Metabolomes task group of the International Metabolomics Society has issued a call to arms to identify and map all metabolites onto metabolic pathways and to relate these pathways across multiple species within the context of evolutionary metabolomics (or phylometabolomics) [8]. The scale of this endeavour means that the group has prioritized the deep investigation of established model organism metabolomes in microbial, plant, and animal biology, promising an avalanche of new metabolic data. Exponential growth is observed in biological databases, and MetaboLights is no exception (Fig. 1).

Metabolomics datasets submitted to MetaboLights contain lists of metabolites that have been identified in those respective studies for a given species in a given biological context. This steady stream of assigned metabolites, together with species and organism part information, is leading to an evidence-based assembly of metabolomes for species, with more complete annotations for the model organisms under investigation worldwide. We believe that this submission-driven assembly, backed by automated and manual quality control, is the only sustainable model for large-scale species metabolome assembly and that it will lead to an indispensable knowledge base for chemical biology research. This common framework is also essential to provide the crystallization point to initiate cross-species to cross-division metabolic analysis of commonality and uniqueness.

**Figure 2:**
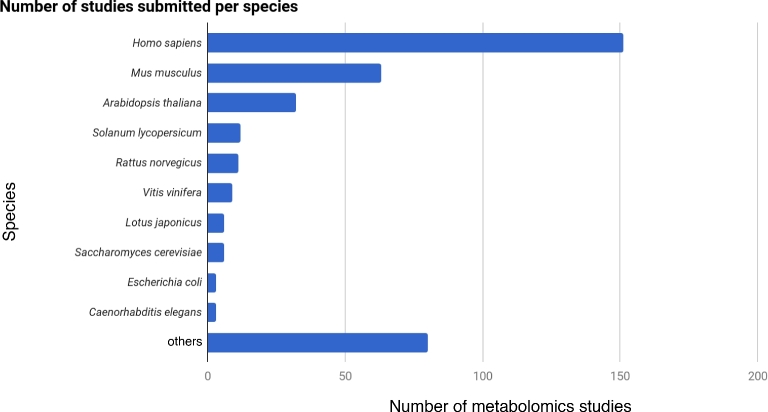
Bar chart distribution of the number of studies in MetaboLights by species. The distribution reflects the most used model species in biological and biomedical research.

Studies in MetaboLights are created by researchers in ISA-Tab format, by either automatically creating datasets from inhouse laboratory information management systems (rare) or manually creating ISA-Tab archives with the help of the ISA tools suite (common). Naturally, the species coverage of studies follows the preferences for model species around the globe (Fig. 2).

The key to this process is the application of online ontologies from BioPortal, combined with local controlled vocabularies to ensure correct terms are used to describe biological samples and experimental factors. Assignment of identified metabolites is done in Metabolite Identification Files, a bespoke extension to the ISA suite.

When the submitter has completed the annotations and the study satisfies all mandatory validations, the study is flagged as ready for curation [9]. At this stage, the curation team makes any required changes, and the study is ready for review. Journal reviewers are then given a unique URL to access the complete study. When the journal review process is complete, the study can be made publicly accessible. The traditional model of curated chemical databases scales linearly, both with time and the number of curators involved in the database assembly.

In contrast, the MetaboLights reference layer grows via a 2-tiered approach for assembling metabolome information. We collect historical information about metabolites found in species from the primary literature and link it with the experimental annotations submitted to MetaboLights, thus generating many of the data points for common and rarer species. In a similar manner as before, this manually curated data process grows linearly with the number of curators working on it.

The second source for metabolome information is submissions from the community, triggered by their commitment to provide open access data, and/or the requirement from funders and publishers to deposit data in an open and accessible manner. The sustainability and efficiency of this second tier is the key argument of this article.

**Figure 3:**
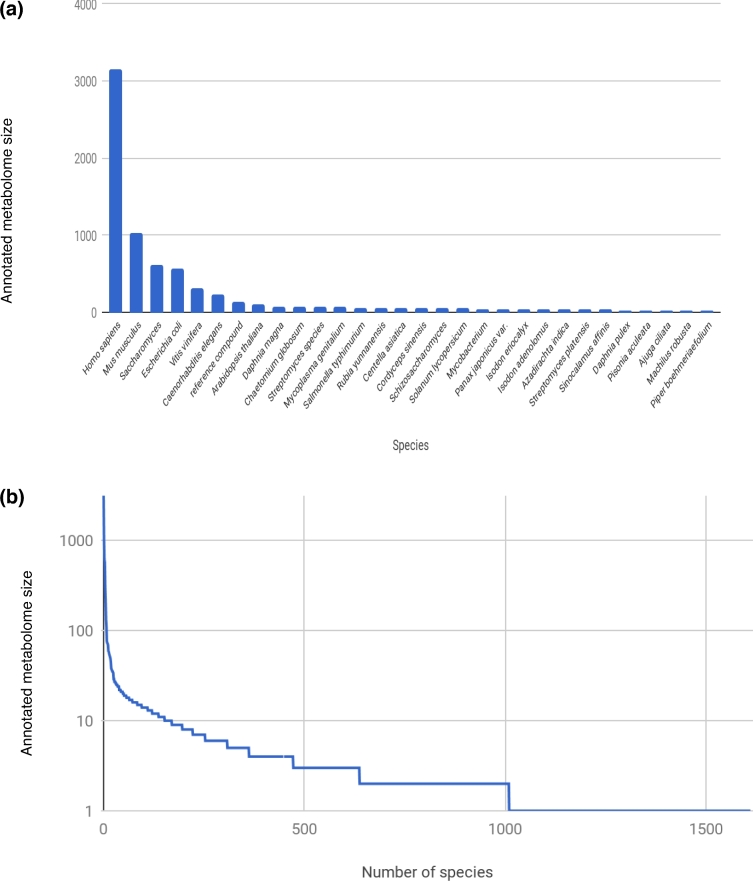
Long-tail distribution of metabolites per species in the MetaboLights reference layer. A few model species are covered very well, while for the majority of more than 1600 species, only a few metabolites were reported. These data cover both metabolites reported in studies and those manually added from the literature by MetaboLights and ChEBI curators. a) Truncated version with the 30 most annotated species. b) Full graph.

Figure 3 shows the current distribution of metabolites per species in the MetaboLights reference layer. Sorted by frequency, this shows a typical long-tail distribution; a few model species are well covered, while only a few metabolites are available for most species. These data include both metabolites reported in studies and those manually curated by MetaboLights and Chemical Entities of Biological Interest (ChEBI) from the literature.

## Conclusion

We have established a model in which information about metabolites in species metabolomes grows dynamically through submissions to public archives such as the MetaboLights database. For the first time, this will automatically provide both the information about which metabolites are found in which species and the supporting evidence—the primary spectroscopic data and supporting meta-data—in a community-driven way. In turn, this will provide up-to-date knowledge bases for fields such as chemical biology, metabolomics, and biomedicine.

## Availability of data and materials

Data underlying the analysis presented here are available without restrictions in the MetaboLights database (RRID:SCR_014663) [5].

## Abbreviations

ChEBI: Chemical Entities of Biological Interest; EMBL-EBI: European Molecular Biology Laboratory-European Bioinformatics Institute.

## Competing interests

None of the authors has any competing interests.

## Funding

The development of MetaboLights was funded by the Biotechnology and Biological Sciences Research Council (BBSRC; http://dx.doi.org/10.13039/501100000268, grant numbers BB/I000933/1 and BB/L024152/1).

## Author contributions

R.M.S., P.C., K.C., K.H., M.W., N.K., P.M., K.J., J.R.M., and V.C.N. developed and curated the MetaboLights database. R.D.H., L.K., M.R.V., C.O.D., and C.S. conceived this study and performed the analysis. All authors have read and approved the manuscript.

## Supplementary Material

GIGA-D-17-00158_Original-Submission.pdf

GIGA-D-17-00158_Revision-1.pdf

Response-to-Reviewer-Comments_Original-Submission.pdf
