# Supplementary material for: Automated assembly of species metabolomes through data submission into a public repository
Source: Gigascience. 2017 Jul 19;6(8):1–4. doi: 10.1093/gigascience/gix062 (PMC5737527; doi:10.1093/gigascience/gix062)
Supplement: GIGA-D-17-00158_Original-Submission.pdf [file gix062_giga-d-17-00158_original-submission.pdf]

Manuscript

Click here to download Manuscript AutomatedAssemblyofSpeciesMetabolomes.pdf

Automated Assembly of Species Metabolomes through Data Submission into a Public Repository

Reza M Salek[1], Pablo Conesa[1], Keeva Cochrane[1], Kenneth Haug[1], Mark Williams[1], Namrata Kale[1], Pablo Moreno[1], Kalai Jayaseelan[1], Jose Ramon Macias[1], Venkata Chandrasekhar[1] Nainala, Robert D. Hall[4], Laura K. Reed[2], Mark R. Viant[3], Claire O'Donovan[1] and Christoph Steinbeck[1,5,*]

[1] European Molecular Biology Laboratory, The European Bioinformatics Institute (EMBL-EBI), Wellcome Genome Campus, Hinxton, Cambridge CB10 1SD UK

[2] Department of Biological Sciences, University of Alabama, Tuscaloosa, AL 35487 USA

[3] School of Biosciences, University of Birmingham, Birmingham B15 2TT UK

[4] Wageningen University & Research, Wageningen Plant Research - Bioscience, P.O. Box 16, 6700AA, Wageningen, The Netherlands

[5] Institute for Inorganic and Analytical Chemistry, Friedrich-Schiller-University, 07743 Jena, Germany

ABSTRACT

Following similar global efforts to exchange genomic and other biomedical data, we have now witnessed the emergence of global databases in metabolomics. The MetaboLights database, the first general purpose, publically-available, cross-species, cross-application database in metabolomics, has become the fastest growing data repository at the EMBL-EBI in terms of data volume. Here we present the automated assembly of species metabolomes in MetaboLights, a crucial reference for chemical biology, growing through user submissions.

Background

Following data standardisation efforts in the 1990s and the success of global efforts to exchange genomic [1] [2], proteomic [3], gene expression [4] and other biomedical data, we have now witnessed the emergence of global databases in metabolomics. In 2012, the European Bioinformatics Institute (EMBL-EBI) launched the MetaboLights database (RRID:SCR_014663) [5], the first general purpose, cross-species, cross-application database in metabolomics, aiming at a similar growth in this remaining large pillar of omics sciences [6]. Within the first two years after its inception, MetaboLights became the fastest growing data repository at the EMBL-EBI in terms of data volume. Here we present the automated assembly and growth of species metabolomes in the MetaboLights reference layer, which is largely driven by user submissions. Following the Bermuda principles[7], which led to the mandatory deposition of data in repositories such as the European Nucleotide Archive [2] or GenBank [1], a number of journals and publishers already demand or recommend the deposition of metabolomics studies in MetaboLights. These include the Nature Journals,

including, of course, this journal, the EMBO journal, PLOS journals, MDPI *Metabolites*, BioMed Central, Frontiers journals and *Metabolomics*. To the best of our knowledge, MetaboLights is the only global, general purpose repository which systematically requires the submission of a metabolites assignment - a requirement which is fundamental for the process described here.

Findings

One of the fundamental, unsolved problems in Metabolomics is the availability of exhaustive model organism metabolomes. The newly formed Model Organism Metabolomes task group of the international Metabolomics Society has issued a call to arms to identify and map all metabolites onto metabolic pathways and to relate these pathways across multiple species within the context of evolutionary metabolomics (or phylometabolomics)[8]. Due to the scale of this endeavour, the group has prioritised the deep investigation of established model organism metabolomes in microbial, plant and animal biology, promising an avalanche of new metabolic data. Exponential growth is observed in biological databases and MetaboLights makes no exception (Figure 1).

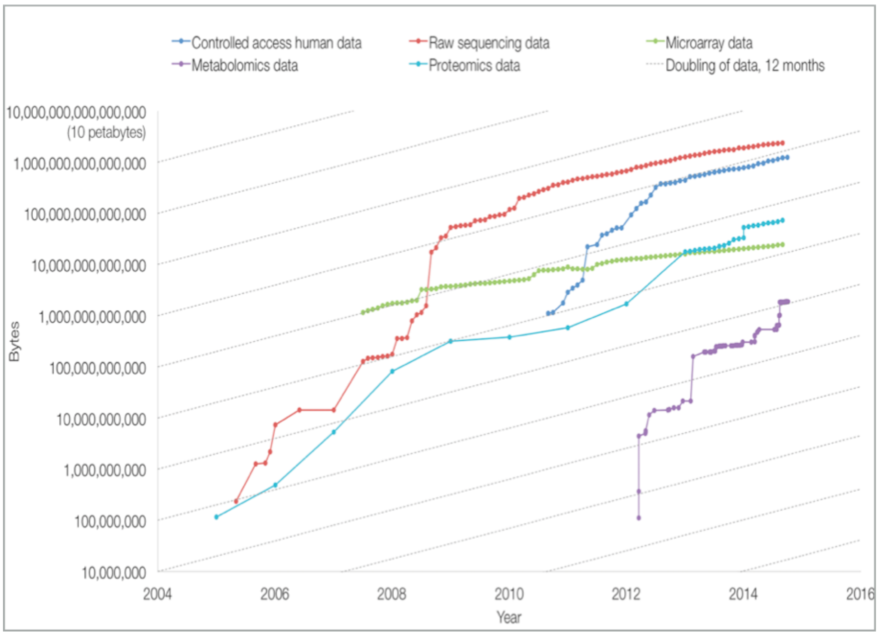

Figure 1: Growth in data repositories at the European Bioinformatics Institute (EMBL-EBI). The graph shows the data volume in each of the repositories over time on a logarithmic scale. Shown are repositories for controlled access human data, raw sequencing data, microarray, proteomics and metabolomics data. Archives were started at different moments in history. Metabolomics shows the steepest growth of all repositories at EMBL-EBI.

Metabolomics datasets submitted to MetaboLights contain lists of metabolites that have been identified in those respective studies for a given species in a given biological context. **This steady stream of assigned metabolites together with species and organism part information is currently leading to an evidence-based assembly of metabolomes for species, with more complete annotations for the model organisms under investigation worldwide.** We believe that this submission driven assembly, backed by automated and manual quality control, will be the only sustainable model for large scale species

metabolome assembly and will lead to an indispensable knowledge base for chemical biology research. This common framework is also essential to provide the crystallization point for initiating cross-species to cross-division metabolic analysis of commonality and uniqueness.

Studies in MetaboLights are created by researchers in ISA-TAB format [9], using either automatic creation of datasets from inhouse LIMS systems (rare) or manual creation of ISA-TAB archives with the help of the ISA-tools suite (common). Naturally, the species coverage of studies follows the preferences for model species around the globe (Figure 2).

Key to this process is the application of online ontologies from BioPortal, combined with local controlled vocabularies to ensure correct terms are used to describe biological samples and experimental factors. These controlled vocabularies include the NCBI taxonomy [10] for species identification, the BRENDA tissue ontology [11] and the Experimental Factor Ontology (EFO) [12]. The assignments of identified metabolites is done in Metabolite Identification Files (MAF), a bespoke extension to the ISA suite.

When the submitter has completed the annotations and the study satisfies all mandatory validations, the study is flagged as ready for curation [13]. At this stage the curation team will make any required changes, enabling the study to be ready for review. Journal reviewers are then given a unique URL to access the complete study. After the journal review process completes, the study is ready to be publicly accessible. The traditional model of curated chemical databases scales linearly both with time and the number of curators involved in the database assembly.

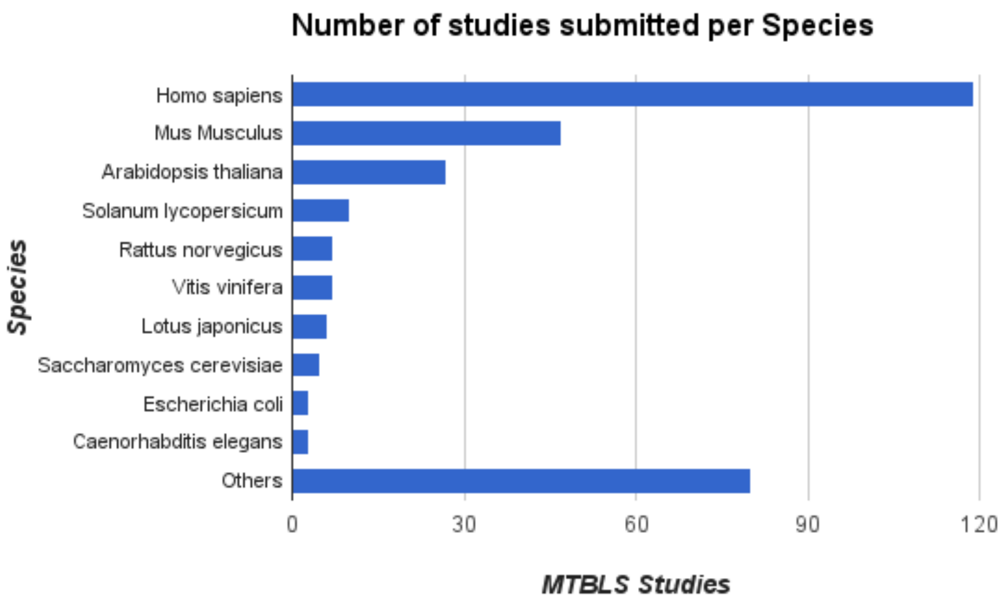

Figure 2: Bar chart distribution of Number of studies in MetaboLights by species. The distribution is reflecting the most used model species in biological and biomedical research.

In contrast, the MetaboLights reference layer grows through a two-tier approach for assembling metabolome information. We collect historical information about metabolites found in species from the primary literature, linking it with the experimental annotations submitted to the MetaboLights, thus generating many of the data points for common and rarer species. In a similar manner as before, this manually curated data process grows linearly with the number of curators working on it.

The second source for metabolome information are submissions from the community triggered by their commitment to open access data and/or the requirement from funders and publishers to deposit data in an open and accessible manner. The sustainability and efficiency of this second tier is the key argument of this article.

Figure 3 shows the current distribution of metabolites per species in the MetaboLights reference layer. Sorted by frequency, this shows a typical long-tail distribution with a few model species being well covered, while for the majority of species, only a few metabolites are available. This data includes both metabolites reported in studies as well as those manually curated by MetaboLights and ChEBI curators from the literature.

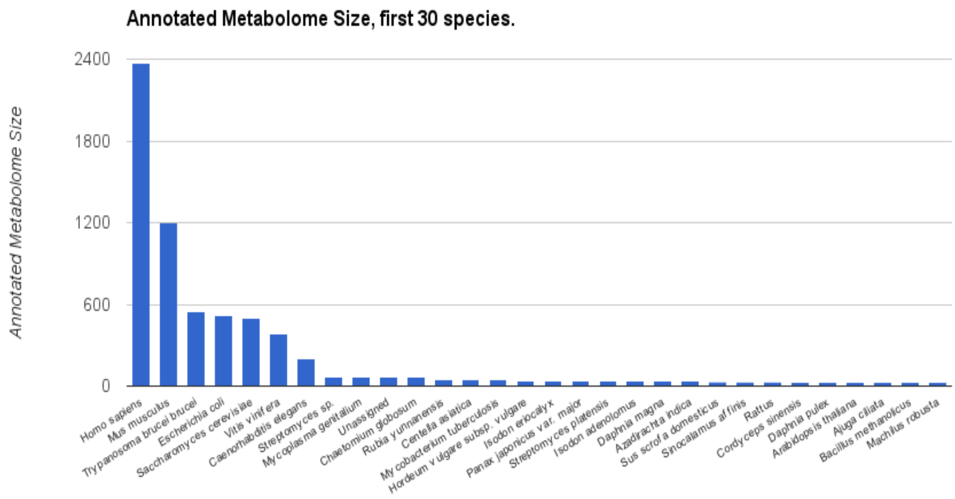

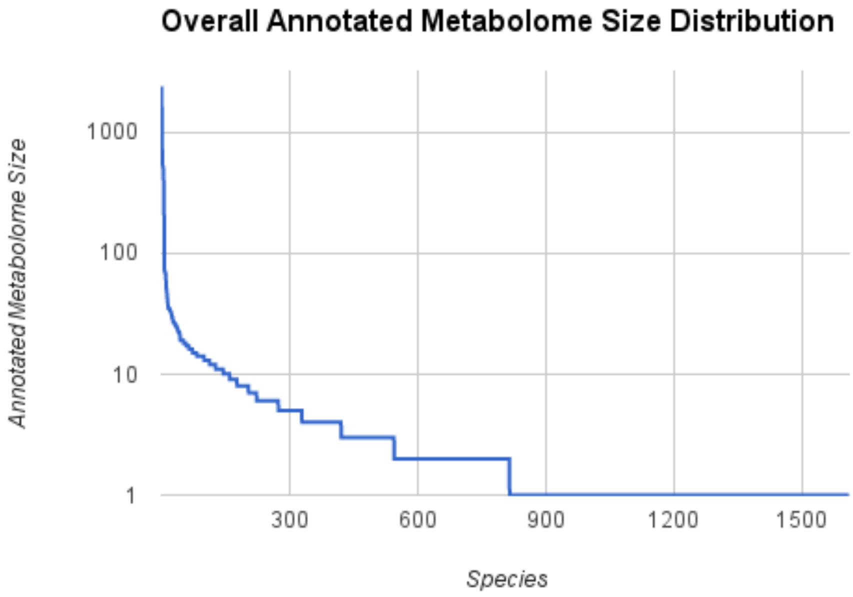

Figure 3: Long-tail distribution of metabolites per species in the MetaboLights reference layer. A few model species are covered very well, while for the majority of over 1600 species, only a few metabolites were reported. This data covers both metabolites reported in studies

as well as those manually added from the literature by MetaboLights and ChEBI curators. a) Truncated version with the 30 most annotated species. b) Full graph.

Conclusion

In conclusion, we have established a model, where information about metabolites in species metabolomes grows dynamically through submissions to public archives such as the MetaboLights database. For the first time, this will automatically provide both the information about which metabolites are found in which species and also the supporting evidence - the primary spectroscopic data and supporting meta-data - in a community driven way, providing up-to-data knowledge bases for fields such as chemical biology, metabolomics and biomedicine.

Availability of Data and Materials

All data underlying the analysis presented here is available without restrictions at http://www.ebi.ac.uk/metabolights (RRID:SCR_014663).

Competing Interests

None of the authors have any competing interests

References:

1. Benson DA, Karsch-Mizrachi I, Lipman DJ, Ostell J, Sayers EW. GenBank. Nucleic Acids Res. 2009;37:D26–31.

2. Silvester N, Alako B, Amid C, Cerdeño-Tárraga A, Cleland I, Gibson R, et al. Content discovery and retrieval services at the European Nucleotide Archive. Nucleic Acids Res. Oxford Univ Press; 2015;43:D23–9.

3. Vizcaíno JA, Deutsch EW, Wang R, Csordas A, Reisinger F, Ríos D, et al. ProteomeXchange provides globally coordinated proteomics data submission and dissemination. Nat. Biotechnol. 2014;32:223–6.

4. Kolesnikov N, Hastings E, Keays M, Melnichuk O, Tang YA, Williams E, et al. ArrayExpress update--simplifying data submissions. Nucleic Acids Res. 2015;43:D1113–6.

5. Haug K, Salek RM, Conesa P, Hastings J, de Matos P, Rijnbeek M, et al. MetaboLights—an open-access general-purpose repository for metabolomics studies and associated meta-data. Nucleic Acids Res. [Internet]. 2012; Available from: http://nar.oxfordjournals.org/content/early/2012/10/28/nar.gks1004.abstract

6. Steinbeck C, Conesa P, Haug K, Mahendraker T, Williams M, Maguire E, et al. MetaboLights: towards a new COSMOS of metabolomics data management. Metabolomics. Springer; 2012;8:757–60.

7. Schofield PN, Bubela T, Weaver T, Portilla L, Brown SD, Hancock JM, et al. Post-publication sharing of data and tools. Nature. 2009;461:171–3.

8. Edison AS, Hall RD, Junot C, Karp PD, Kurland IJ, Mistrik R, et al. The Time Is Right to Focus on Model Organism Metabolomes. Metabolites [Internet]. 2016;6. Available from: http://dx.doi.org/10.3390/metabo6010008

9. Sansone S-A, Rocca-Serra P, Field D, Maguire E, Taylor C, Hofmann O, et al. Toward interoperable bioscience data. Nat. Genet. 2012;44:121–6.

10. Federhen S. The NCBI Taxonomy database. Nucleic Acids Res. Oxford Univ Press; 2012;40:D136–43.

11. Gremse M, Chang A, Schomburg I, Grote A, Scheer M, Ebeling C, et al. The BRENDA Tissue Ontology (BTO): the first all-integrating ontology of all organisms for enzyme sources. Nucleic Acids Res. 2011;39:D507–13.

12. Malone J, Holloway E, Adamusiak T, Kapushesky M, Zheng J, Kolesnikov N, et al. Modeling sample variables with an Experimental Factor Ontology. Bioinformatics. Oxford Univ Press; 2010;26:1112–8.

13. Salek RM, Haug K, Conesa P, Hastings J, Williams M, Mahendraker T, et al. The MetaboLights repository: curation challenges in metabolomics. Database . 2013;2013:bat029.
