# Supplementary material for: Automated assembly of species metabolomes through data submission into a public repository
Source: Gigascience. 2017 Jul 19;6(8):1–4. doi: 10.1093/gigascience/gix062 (PMC5737527; doi:10.1093/gigascience/gix062)
Supplement: Response-to-Reviewer-Comments_Original-Submission.pdf [file gix062_response-to-reviewer-comments_original-submission.pdf]

Author's Response To Reviewer Comments

We thank the editors for their valuable suggestions and most importantly the copy editor for her help to improve the manuscript. We have implemented all requested changes.
